# Gut Microbiota in Heart Failure—The Role of Inflammation

**DOI:** 10.3390/biomedicines13040911

**Published:** 2025-04-09

**Authors:** Petros N. Fountoulakis, Panagiotis Theofilis, Panayotis K. Vlachakis, Paschalis Karakasis, Konstantinos Pamporis, Marios Sagris, Yannis Dimitroglou, Panagiotis Tsioufis, Evangelos Oikonomou, Konstantinos Tsioufis, Dimitris Tousoulis

**Affiliations:** 1 1st Department of Cardiology, Hippokration General Hospital, National and Kapodistrian University of Athens, 11527 Athens, Greece; pfountou@yahoo.gr (P.N.F.); panos.theofilis@hotmail.com (P.T.); vlachakispanag@gmail.com (P.K.V.); konstantinospab@gmail.com (K.P.); masagris1919@gmail.com (M.S.); dimiyann@hotmail.com (Y.D.); ptsioufis@gmail.com (P.T.); ktsioufis@gmail.com (K.T.); 2 2nd Department of Cardiology, Hippokration General Hospital, Aristotle University of Thessaloniki, 54642 Thessaloniki, Greece; pakar15@hotmail.com; 3 3rd Department of Cardiology, Thoracic Diseases General Hospital “Sotiria”, National and Kapodistrian University of Athens, 11527 Athens, Greece; boikono@gmail.com

**Keywords:** heart failure, gut microbiome, inflammation, short-chain fatty acid, trimethylamine N-oxide, NLRP3 inflammasome

## Abstract

Heart failure (HF) has become an immense health concern affecting almost 1–2% of the population globally. It is a complex syndrome characterized by activation of the sympathetic nervous system and the Renin–Angiotensin–Aldosterone (RAAS) axis as well as endothelial dysfunction, oxidative stress, and inflammation. The recent literature points towards the interaction between the intestinal flora and the heart, also called the gut–heart axis. The human gastrointestinal tract is naturally inhabited by various microbes, which are distinct for each patient, regulating the functions of many organs. Alterations of the gut microbiome, a process called dysbiosis, may result in systemic diseases and have been associated with heart failure through inflammatory and autoimmune mechanisms. The disorder of intestinal permeability favors the translocation of microbes and many metabolites capable of inducing inflammation, thus further contributing to the deterioration of normal cardiac function. Besides diet modifications and exercise training, many studies have revealed possible gut microbiota targeted treatments for managing heart failure. The aim of this review is to demonstrate the impact of the inflammatory environment induced by the gut microbiome and its metabolites on heart failure and the elucidation of these novel therapeutic approaches.

## 1. Introduction

Heart failure (HF) has become an immense health concern affecting almost 1–2% of the population globally [1]. The lifetime risk of HF approaches up to 25%, while its community-wide mortality rate may be exacerbated by the ongoing aging of the population [2]. Although established treatments have ameliorated survival from 1 to 6 years, the prognosis of these patients remains poor, with severe morbidity and mortality, low quality of life, and additional socioeconomic health burden [3].

**Figure 1:**
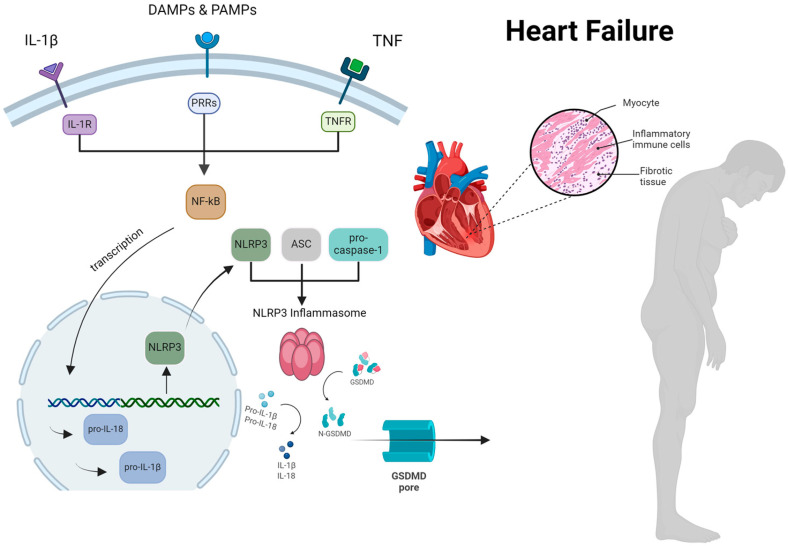
The comprehensive process by which the NLRP3 inflammasome activation occurs, resulting in myocardial cell hypertrophy and inflammation in patients with heart failure. ASC, apoptosis-associated speck-like protein containing a CARD; DAMP, damage-associated molecular patterns; GSDMD, gasdermin D; IL, interleukin; NF-kB, nuclear factor k-light-chain enhancer of activated B cells; NLRP3, nucleotideoligomerization domain (NOD)-like receptor P3; PRRs, pattern recognition receptors; PAMP, pathogen-associated molecular patterns; TNF, tumor necrosis factor. Reproduced with permission from Vlachakis PK et al. [9], *International Journal of Molecular Sciences*; published by MDPI, 2024, and used under Creative Commons CC BY 4.0 license.

HF is defined as a complex clinical syndrome resulting from any structural or functional abnormality impairing cardiac filling and/or blood pumping [4]. As a result, activation of the sympathetic nervous system, the Renin–Angiotensin–Aldosterone (RAAS) axis, endothelial dysfunction, oxidative stress, and inflammation may contribute to the chronic deterioration of cardiac function [5]. Among these mechanisms, inflammation plays a pivotal role in the evolution of HF, which is mostly mediated by several pro-inflammatory cytokines, such as increased levels of tumor necrosis factor-α (TNF-a), interleukin-1 (IL-1), interleukin-6 (IL-6), interleukin-10 (IL-10), interleukin-17 (IL-17), interleukin-18 (IL-18) and reduced concentrations of interleukin-5, (IL-5), interleukin-7 (IL-7), and interleukin-33 (IL-33) [6,7]. The role of the NLRP3 inflammasome is critical in this regard, as it is considered the orchestrator of inflammation in HF (Figure 1). It is widely known that chronic inflammatory disorders (e.g., arterial hypertension, diabetes mellitus, hyperlipidemia, ischemic cardiomyopathy, atrial fibrillation, chronic kidney disease, asthma/ chronic obstructive pulmonary disease, depression) as well as metabolic factors (e.g., obesity and metabolic syndrome) and lifestyle behaviors (e.g., smoking, physical inactivity, alcohol abuse) are regarded as significant contributors to the progression of HF [8].

Because the etiology of inflammation in HF has not been totally understood, recent studies have focused their interest on the gut microbiome. The gastrointestinal tract is always inhabited by many microorganisms, whose genome, transcription, and translation products are responsible for numerous vital functions of our organism [10]. However, dysfunction of the intestinal microbiome, a process called dysbiosis, has been associated with increased cardiovascular risk [11]. Dysbiosis also favors the production of toxic metabolites through inflammatory pathways, which may induce or worsen preexisting coronary artery disease, hypertension, diabetes mellitus, hyperlipidemia, and HF [12,13,14]. However, these diet-related traditional cardiovascular risk factors may induce a chronic inflammatory environment, which may result not only in the alteration of the gut microbiome but also its epigenetic inheritance to descendants [15].

Growing evidence reveals an interrelationship between gut dysbiosis and HF, which is mediated by inflammation. The most prominent theory supporting the induction of HF refers to the “leaky gut” hypothesis [16]. Gut dysbiosis may be the result of (i) the reduction of intestinal bacterial diversity, (ii) the overproduction of harmful bacteria, and (iii) loss of the beneficial bacterial gut population [17]. The two most common mechanisms impairing the intestinal population in HF include reduced cardiac output and overactivity of the sympathetic nervous system, thus deteriorating the natural function of the intestinal wall barrier through vasoconstriction [18]. The pathophysiologic pathways differ depending on overall systolic cardiac function. Therefore, the disruption of the intestinal barrier permeability wall favors bacterial translocation and the diffusion of toxic bacterial compounds into systemic circulation through the TLR4/NF-κB activation pathway and the NLRP3 inflammasome, resulting in sub-acute or chronic inflammation [19]. Continuous exposure to systemic inflammation accelerates the evolution of HF. Consequently, gut dysbiosis and HF interact bidirectionally as inflammation may aggravate equally each of these two entities.

Τhis interaction between the gut microbiome and HF has progressively triggered scientific interest and can be characterized as an emerging field of study for the discovery of novel HF treatments [14]. The aim of this review is to demonstrate the impact of the inflammatory environment induced by the gut microbiome and its metabolites on HF and the elucidation of these novel molecular targets.

## 2. Gut Microbiota

Our gastrointestinal tract abounds with trillions of microbes, which mostly include bacteria and viruses [20]. The normal gut flora may contain more than 3 million genes, compared to the almost 23,000 genes of the human genome, demonstrating the significant impact of these species on human health [21]. The genetic information and the functions of these microbes are described as gut microbiomes.

According to recent studies, the community of the adult intestinal tract is mostly dominated by *Bacteroidetes, Firmicutes*, and, to a lesser extent, *Actinobacteria, Proteobacteria, Verrucomicrobia, Cyanobacteria*, and *Eukarya* [22]. In particular, the small intestine is mostly colonized by Gram-positive bacteria of the phylum *Firmicutes*, which comprise the classes of *Streptococcus, Veillonella, and Clostridium* [23]. However, the large intestine has greater microbial diversity, with domination of the phyla of *Bacteroidetes* (class of *Bacteroides*) and *Firmicutes* (class of *Clostridium, Faecalibacterium, Lactobacillus* and *Ruminococcus*) [24]. The composition of the bacterial community colonizing the gastrointestinal tract may be influenced mainly by eating habits and much less by other factors, such as the method of delivery, geographic origin, host genes, age, gender, body mass index (BMI), and treatment with antibiotics and/or probiotics [25]. It is noteworthy that *Bacteroidetes, Firmicutes*, *Actinobacteria*, and *Proteobacteria* may be affected by numerous factors in our everyday life, including, in particular, overuse of chlorinated water, consumption of food additives, and exposure to pollutants, such as mycotoxins, heavy metals, pesticides, and antibiotics [26]. Dysregulation of the residing *Verrucomicrobia* population, especially *Akkermensiamuciniphila*, could reduce their efficacy in restraining inflammatory bowel disease (IBD) and obesity and may modify the intestinal environment, thus increasing the risk of carcinogenesis [27]. Additionally, the imbalance of the Eukarya, especially yeasts, a minor portion of the intestinal flora, is associated with certain autoimmune diseases, such as irritable bowel syndrome (IBS), as well as diabetes and colorectal cancer [28]. Exposure to the inflammatory effect of the Cyanobacterial toxin may result in neurodegenerative pathologies, including Parkinson’s disease, amyotrophic lateral sclerosis (ALS), and Alzheimer’s disease [29].

The interaction between the intestinal microbiome and the gastrointestinal tract is not only symbiotic but also beneficial for human health. The human body (host) is responsible for the growth and protection of the intestinal bacteria, while these, in turn, facilitate digestion, the production of vitamins, the inhibition of pathogen overgrowth and colonization, and the regulation of the immune response. The significance of the intestinal microbiome in overall health and longevity has been proven recently, although Hippocrates highlighted the importance of a healthy digestive system [30]. This symbiotic interaction is moderated during different stages of life [31].

The main functions of the intestinal microbiome in human health include the following:

1. Nutrient metabolism
  The intestinal microbiota produces a variety of important nutrients for human health. In particular, a significant amount of nutrients is received from carbohydrates, which are either absorbed directly or firstly digested and then absorbed as simple sugars in the upper parts of the gastrointestinal (GI) tract. Members of *Bacteroides* are mostly involved in carbohydrate metabolism [32]. A portion of these ingested carbohydrates escapes digestion in the upper gastrointestinal tract and is redirected for further fermentation by microbial populations, such as *Bacteroides, Roseburia, Bifidobacterium, Fecalibacterium, and Enterobacteria* [33]. The products are gases and short-chain fatty acids, such as butyric, acetic, and propionic acid, resulting from the interaction of the ligand–receptor of SCFAs with a G-protein-coupled receptor, Gpr41, representing important energy resources for the host [34].
  Proteins also remain an essential part of the human diet, which are mostly metabolized by the host’s digestive enzymes. The gut microbiome retains effective protein metabolism through microbial proteinases and peptidases as well as human proteinases [35]. Another important function that should be emphasized is the synthesis of vitamins K and B (such as B1 or “thiamine”, B2 or “riboflavin”, B3 or “niacin”, B5 or “pantothenic acid”, B6 or “pyridoxine”, B7 or “biotin”, B9 or “folic acid”, and B12 or “cobalamin”) [36]. Members of the genus *Bacteroides* also produce conjugated linoleic acid, an agent with antiatherogenic, anticancer, hypolipidemic, and immunomodulatory properties [37].
  Regarding lipids, the intestinal microbiome suppresses lipoprotein lipase activity in adipocytes. It is noteworthy that the *Bacteroides thetaiotaomicron* stimulates lipid hydrolysis in these tissues [38]. The most important fatty acids produced by the fermentation of plant fibers include the following. I. Acetic acid, which regulates appetite and lipid and cholesterol metabolism. II. Butyric acid, which produces energy, especially for colon cells, regulates glucose metabolism, and contributes to insulin resistance and cancer cell apoptosis. III. Propionic acid, which may affect the regulation of appetite and glucose metabolism [39]. Short-chain fatty acids generally reduce the risk of obesity and diabetes and empower the immune system or even favor brain development [40]. Interestingly, the study by Firrman et al. on animal models revealed that changes in the environmental pH resulted not only in an altered intestinal microbiome but also reduced pH, which was accompanied by significantly reduced levels of short-chain fatty acids (SCFA) [41].
  In 2009, Fukiya et al. demonstrated the conversion of primary bile acids into secondary bile acids and deoxycholic and lithocholic acids in the human colon by *Bacteroidetes intestinalis, Bacteroidetes fragilis* and *Escherichia coli* [42]. In conclusion, the intestinal microbiome plays a significant role in nutrient metabolism and balance within the host organism.
2. Regulation of the immune response and antimicrobial protection
  The gut microbiome of a healthy individual is in constant interaction with the native immune system. This homeostatic balance includes “immunological tolerance” against the host’s antigens and an effective immune response against harmful agents. The factors participating in the immunomodulatory process include the Gut Associated Lymphoid Tissue (GALT), T lymphocytes (CD4 and CD8), and IgA produced by plasma cells, as well as local macrophages and dendritic cells [43]. The immune system constantly supervises the intestinal flora by preventing their overgrowth through mechanisms like mucus production by goblet cells, the secretion of antimicrobial peptides by Paneth cells, and the production of IgA by B-cells [44,45]. Disturbance of this balance may increase the risk of infection, inflammation, cancer, and cardiovascular events.
  The mucus layer of the intestinal mucosa is described as the first barrier against pathogens. A layer of anaerobic bacteria covers the intestinal mucus layer, protecting epithelial colon cells from adhesion and overgrowth of aerobic Gram-negative bacteria. When intestinal dysbiosis occurs, this protective layer is destroyed, facilitating pathogen translocation into systemic circulation [46]. Epithelial cells also contribute to the immune response of the intestinal mucosa, not only directly but also through the secretion of cytokines and chemokines from the intestinal mucosa [47].
  The balance of the intestinal microbiome and the immune system reveals the presence of a least-inflammatory environment, which is capable of perceiving signals emitted by the inhabiting intestinal flora of the host. These signaling markers, which include liposaccharides, peptides, and peptidoglycan, are referred to as pathogen-associated molecular patterns or PAMPS and are recognized through specific pathways [48].
  When imbalance of the intestinal barrier occurs, pathogens will submit to the process of phagocytosis induced by tissue macrophages. Activation of the autophagy pathway affects Paneth cell function, increases IL-1β production, and promotes the activation of regulatory T-cells (T-reg) and IgA production by B lymphocytes [49]. MYD88 may mediate ΙgA secretion by B-lymphocytes of the intestinal epithelium through activation of nuclear factor–kappa–B (Nf-κB), which controls the immune and inflammatory response of the host organism [50].
  The gut microbiome may also affect neutrophil migration and their differentiation into macrophages, the differentiation of the T cell population into Th1, Th2, and Th17, which promote inflammation, as well as the function of regulatory T cells (T-regs), which restrict the inflammatory process [51,52].
  Innate lymphoid cells (ILCs) located on the intestinal epithelium may produce cytokines and exert an immunomodulatory effect on inflammation. ILCs can be divided into three groups: group 1/T-bet +, group 2/GATA-3 +, and group 3/RORγt + [53]. IECs release an enzyme called “intestinal alkaline phosphatase”, which dephosphorylates the endotoxin LPS and may reduce neutrophil migration through the gastrointestinal tract [54].
  In conclusion, a healthy intestinal microbiome contributes to the preservation of the intestinal barrier’s function, thus inhibiting the entrance of harmful microbes or metabolites into systemic circulation. Through this continuous interaction, the immune system acquires an effective defense system against numerous pathogens, contributing to the maintenance of the host’s homeostasis.
3. Maintenance of the gut barrier’s integrity
  Numerous studies have depicted the pivotal role of the intestinal microbiome in the integrity of the intestinal barrier and the protection of the gastrointestinal tract. Firstly, the *Bacteroides thetaiotaomicron* bacterium triggers the expression of a 2A protein, which is useful for the protection of desmosomes on the intestinal villi [55]. Mechanisms that also contribute to the maintenance of the gut microbiome barrier’s function include toll-like receptor 2 (TRL2) signaling, mediated by the peptidoglycan of the microbial cell wall and the endocannabinoid system [56,57]. According to Matar et al., a high-fat diet may increase the permeability of the intestinal barrier, whereas a diet including zinc, fiber, and vitamin D may reduce its permeability [58]. Quite interestingly, Chittimalli et al. have proven the protective effect of angiotensin-(1–7) on the balance of the intestinal barrier and the inhibitory effect on inflammation in the colon of old animal models through recovery of the intestinal stem cell layer and modulation of the residing gut microbiome [59]. A recent study by Lv et al. denoted the importance of maintaining levels of uric acid within normal range, as hyperuricemia favors the overgrowth of microbes, including, in particular, *Bacteroides, Ruminiclostridium, Akkermansiaceae, Burkholderiaceae, Bilophila* and *Parasutterella*, thus disturbing the intestinal barrier [60].
4. Metabolism of drugs and xenobiotics
  Among other functions of the intestinal microbiome, the metabolism of drugs and xenobiotics should also be mentioned. Xenobiotics are defined as substances unnaturally produced by the host and include drugs, environmental toxins, and heavy metals. According to Collins and Patterson, the intestinal microbes may reduce xenobiotic absorption, thus altering their pharmacokinetics and favoring detoxification in the host organism [61]. Drug metabolism is mediated by enzymatic catalysis, among which the most frequent include reduction and hydrolysis, resulting in either drug activation or inhibition [62]. The best-studied case of enzymatic drug modification is digoxin, administered in cases of heart failure and atrial fibrillation. In certain circumstances, the bacterium *Eggerthella lenta*, belonging to the *Actinobacteria* phylum, reduces the bioavailability of digoxin through its conversion to the inactive product, dihydrodigoxin [63]. In 2009, the study by Clayton et al. highlighted the positive effect of the microbial metabolite p-cresol on the metabolism of acetaminophen due to the inhibition of the hepatic sulfotransferases [64]. Additionally, β-glucuronidase, an enzyme derived from the disintegration of the anticancer drug irinotecan, may affect its side effects, such as diarrhea, anorexia, weight loss, and inflammation [65]. Therefore, drugs and xenobiotic metabolism may reveal the possibility of novel therapeutic strategies to treat various diseases in the future.
  Better understanding of these metabolic modifications may hold the key for the discovery of novel HF therapeutic strategies.

## 3. Gut Dysbiosis, Inflammation, and Heart Failure

Alterations in the population or function of the gut microbiome, a process called dysbiosis, may result in pathologies ranging from local gastrointestinal disorders to diseases of the nervous, respiratory, and cardiovascular systems [66]. Dysbiosis may be the consequence of the imbalance between protective and possibly harmful bacteria or a reduction of microbial diversity [17].

It is widely known that the intestinal epithelium is the first line of defense and responsible for a “screening” process of the incoming molecules through specialized cell junctions, known as “tight junctions”. These junctions absorb important nutrients and remove harmful agents (such as toxins). However, in certain circumstances, their function is deteriorated, allowing toxic agents or metabolites to enter the bloodstream directly. This is referred to as “leaky gut” and may induce numerous pathologies, such as seasonal allergic reactions or asthma, digestive disorders or irritable bowel syndrome (IBS), hormonal imbalances (premenstrual syndrome (PMS) or polycystic ovary syndrome (PCOS)), depression, anxiety, autoimmune diseases (rheumatoid arthritis and celiac disease), chronic fatigue syndrome and fibromyalgia, dermatological diseases, such as acne and eczema, and food allergies and intolerances [67].

**Figure 2:**
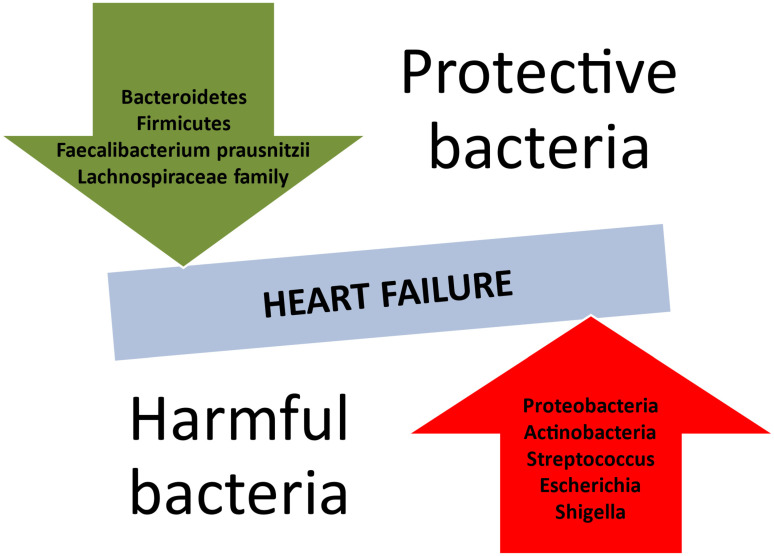
Protective and harmful bacterial species concerning the development of heart failure.

HF predisposes the body to modifications in the composition of the intestinal microbiome (Table 1). The recent literature reveals a reduction in microbial diversity or an absence of crucial gut microbes (Figure 2), such as those producing butyrate, e.g., *Faecalibacterium prasusnitzii*, the *Lachnospiraceae family*, and *Eubacterium hallii* [68,69]. Inflammation-mediated HF is mostly associated with a predominance of Gram-negative bacteria. According to a meta-analysis by Huang et al., *Proteobacteria* and *Actinobacteria* held the lion’s share of the gut microbiota in patients with HF [70]. It is also noteworthy that *Streptococcus* spp., *Escherichia-Shigella*, *Veillonella* sp., *Klebsiella*, and *Enterococcus* were also part of the intestinal community in the HF population [71].

Major differences have been described among the intestinal microbiota, as indicated by the stage of HF measured according to the left ventricular ejection fraction (LVEF). Patients with reduced LVEF had more abundance of *Ruminococcus gnavus* and decreased amounts of *S. Faecalibacterium* [72]. Nevertheless, the GUMPTION Study clearly states that patients with preserved LVEF presented higher levels of *Enterococcus* and *Lactobacillus* and reduced colonies of anti-inflammatory microorganisms (*Butyricicoccus, Sutterella, Lachnospira, Ruminiclostridium*) [73]. Therefore, these patients may be more susceptible to pseudomembranous colitis, most often due to overgrowth of the bacterium *Clostridium difficile* [74].

**Table 1:** Gut microbiota in heart failure—bacterial diversity and its impact.

Bacterial Group	Impact on Heart Failure	Mechanism	Key References
*Bacteroidetes*	Part of normal gut flora; changes linked to dysbiosis	Supports gut barrier integrity; altered composition linked to inflammation and endotoxemia	Huang et al. (2024) [70], Desai et al. (2023) [71]
*Firmicutes*	Reduced in heart failure patients; essential for SCFA production	Produces anti-inflammatory SCFAs (butyrate); depletion leads to inflammation	Cui et al. (2018) [72], Tousoulis et al. (2022) [69]
*Proteobacteria*	Dominant in HF patients; associated with inflammation	Enriched in endotoxin-producing bacteria; triggers NF-κB pathway	Huang et al. (2024) [70]
*Actinobacteria*	Increased in HF patients; correlated with worsened outcomes	Produces metabolites that exacerbate inflammation and oxidative stress	Huang et al. (2024) [70]
*Faecalibacterium prausnitzii*	Anti-inflammatory properties; reduced in HF patients	Produces butyrate; depletion leads to immune dysfunction and inflammation	Cui et al. (2018) [72], Paraskevaidis et al. (2023) [75]
*Lachnospiraceae family*	Decreased levels linked to poor cardiac outcomes	Produces butyrate; supports gut barrier and reduces systemic inflammation	Huang et al. (2024) [70]
*Streptococcus* spp.	Presence associated with increased inflammation	Overgrowth linked to gut permeability and cytokine production	Desai et al. (2023) [71]
*Escherichia-Shigella*	Linked to gut permeability and inflammation in HF patients	Produces endotoxins; triggers TLR4/NF-κB pathway and systemic inflammation	Méndez-Bailón et al. (2020) [74]

It is widely known that inflammation plays a significant role in the pathogenesis of atherosclerotic cardiovascular disease and the evolution of HF. Cytokines are pro-inflammatory proteins produced by the damaged myocardium and include tumor necrosis factor-α, interleukin-1, interleukin-6, and interleukin-18. Their overproduction is enhanced by the excessive stimulation of the sympathetic nervous system. The injured myocardium and reduced cardiac output activate monocytes to release these cytokines. The following cardiomyocyte apoptosis may cause diffuse loss of myocardial tissue and lead to HF [76,77,78]. These factors also disturb cardiac hemodynamic balances, stimulate left ventricular hypertrophy, and induce the secretion of more cytokines. Additionally, tumor necrosis factor α (TNF-α) inhibits cardiac contractility by reducing the production of sarcoplasmic reticulum proteins, thus contributing to the deterioration of cardiac function [79]. Monocyte chemoattractant protein 1 (MCP-1) is a low molecular protein inducing the recruitment and migration of white blood cells, resulting in aggravation of the inflammatory environment [80]. Systemic inflammation favors the extensive collagen deposition of collagen. As a result, collagen becomes more rigid, contributing to the deterioration of both diastolic and systolic cardiac function [81,82].

The interaction between the gut microbiota, inflammation, and HF may be interpreted through the “leaky gut” hypothesis [83]. According to the recent literature, the most common factors favoring the alteration of the gut bacterial population in HF include reduced cardiac output and overdrive of the sympathetic nervous system, deteriorating the natural function of the intestinal wall barrier through vasoconstriction [18]. Cardiac systolic dysfunction, as measured by the left ventricular ejection fraction, may affect differently the inflammatory pathways associating HF with the gut microbiome. HF with reduced ejection fraction (HFrEF) induces hypoperfusion, resulting in ischemia and swelling of the intestinal mucosa. The following progressive visceral congestion disturbs the integrity of the intestinal barrier through which microbes and/or their metabolites may interact and infiltrate the mucosal elements initiating or exacerbating inflammatory processes and immune response. However, HF with preserved ejection fraction (HFpEF), mostly found in patients with metabolic syndrome, may be the consequence of insulin resistance and an increase in adipose tissue caused by the impaired gut microbiome, which provide the elements for inflammation and oxidative stress [84]. Therefore, these persisting mechanisms may favor sub-acute or chronic inflammation, the precursor of HF [75]. When dysbiosis occurs, an imbalance in the composition and function of the intestinal microflora is observed, resulting in modification of the immune response, susceptibility to inflammatory diseases, allergies, and metabolic diseases. Therefore, gut dysbiosis and HF result in an endless loop where each part of the scale aggravates the other through systemic inflammation.

**Figure 3:**
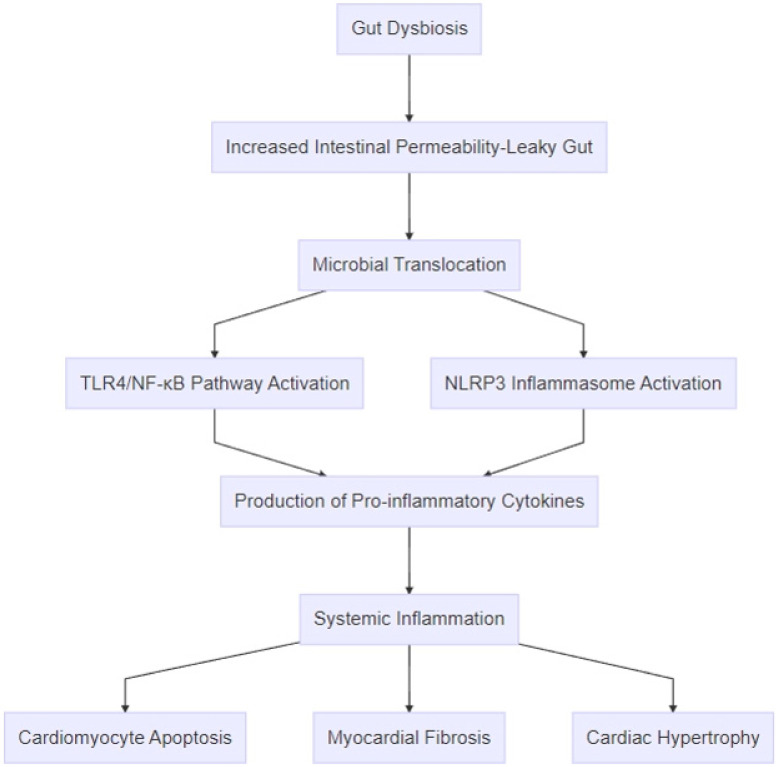
Pathophysiologic mechanisms linking gut dysbiosis to heart failure. The process begins with gut dysbiosis, which leads to increased intestinal permeability (“leaky gut”) and microbial translocation of bacterial components, such as lipopolysaccharides, and metabolites, like trimethylamine-N-oxide. These microbial products activate inflammatory pathways, including the TLR4/NF-κB pathway and the NLRP3 inflammasome, resulting in the production of pro-inflammatory cytokines, such as TNF-α, IL-1β, IL-6, and IL-18. The amplification of systemic inflammation promotes cardiac remodeling through mechanisms like cardiomyocyte apoptosis, myocardial fibrosis, and cardiac hypertrophy.

A growing number of studies have revealed the constant interaction among inflammation, HF, and the gut microbiome (Figure 3). According to Zhang et al., infants with HF related to congenital heart disease presented apart from alterations of the intestinal microbial population predominance of *Enteroccocus*, which may induce the secretion of certain inflammatory mediators, such as interleukin-1β (IL-1β), interleukin-4 (IL-4), interleukin-6 (IL-6), and tumor necrosis factor alpha (TNF-α), thus favoring the evolution of HF [85]. Additionally, the crucial role of inflammation in gut–heart interplay is depicted by fecal calprotectin (FC), an inflammatory biomarker of the intestine produced by monocytes and neutrophils affecting the status of HF, probably through stimulation of the NF-kB e p38 MAPK pathway [86]. Yuzefpolskaya et al. support that changes in the intestinal flora and endotoxinemia were more profound in patients with a left ventricular assist device (LVAD) or heart transplantation (HT), whereas the group with a worsening HF class had more elevated inflammatory and oxidative stress biomarkers [87]. One study on animal models with HF with preserved ejection fraction (HFpEF) suggested that high-protein and high-fat diets resulted in decreased gut diversity, imbalance of the intestinal barrier, and potential translocation of TMAO and endotoxins in systemic circulation capable of generating an inflammatory environment. It is noteworthy that modification of the gut microbiome was occurring simultaneously with the progression of HF [88]. Furthermore, Halade et al. have proven that sleep loss, when combined with excessive dietary fat consumption in post-myocardial infarction (MI) rats, triggered not only changes in the gastro-intestinal microbiome but also an increase in inflammatory prostaglandins and cytokines, such as IL1-β and IL-6, aggravating ongoing HF [89]. Consequently, inflammation remains one of the most important pathophysiologic mechanisms between the gut microbiome and HF.

## 4. The Role of Inflammatory Mediators of the Gut–Heart Axis

The gut–heart axis has become, over the years, a significant notion referring to the mutual interaction between the gut microbiota and cardiovascular diseases through numerous pathways [90]. Among these, inflammation plays a central role in the evolution of HF [9,91]. Therefore, the discovery of novel inflammatory biomarkers is required for a better understanding of this bidirectional association. So far, recent studies have revealed markers, such as endotoxins, trimethylamine n-oxide (TMAO), short-chain fatty acids, zonulin, and amino acids, that may be promising (Table 2).

### 4.1. Lipopolysaccharide

Lipopolysaccharide (LPS), also known as endotoxin, is a significant membrane component of the Gram-negative bacteria, such as *Escherichia coli* and *Salmonella*, inhabiting the gut and may result in pathologies in case of imbalance of the intestinal barrier [102]. LPS has become an important endothelial pro-inflammatory activator through the nuclear factor kappa-B (NF-κB) pathway in the pathogenesis of HF and may modify the prognosis of these patients [103,104]. Interestingly, higher LPS activity has been found more often in patients with decompensated/congestive HF. In fact, Nguyen et al. suggest that almost half of the population hospitalized for acute HF had excessive endotoxin levels and acidosis of the intestinal mucosal cells [92]. LPS has also been identified as one of the most potent triggers of innate immune activation, while other evidence suggests the development of allergy, autoimmune response, and chronic inflammation [105]. Asgharzadeh et al. found that LPS induced in animal models several pro-inflammatory mediators, mainly interleukin 6 (IL-6), tumor necrosis factor-α (TNF-α), and myocardial accumulation of inflammatory cells [93]. Yuzefpolskaya et al. confirmed the presence of oxidative stress and chronic inflammation, especially in advanced HF stages, especially class IV, according to the New York Heart Association (NYHA) functional classification for HF [106]. Under septic conditions, LPS may bind to the CD14/TLR4/MD2 complex, promoting the secretion of pro-inflammatory cytokines and leading to myocardial apoptosis [107]. In addition, Toll-like receptor-4 (TLR-4) activated by LPS is capable of activating the innate immune response through pro-inflammatory cytokines, inflicting myocardial apoptosis and fibrosis and further worsening normal cardiac function [108]. Moreover, Lipid A (or endotoxin molecule) is a lipid element of endotoxin that can initiate inflammatory reactions in the cardiac tissue after binding to TLR-4 [109]. LPS is also a mediator of platelet activation and can influence the coagulation status, aggravating the already existing inflammatory environment [110]. It is worth mentioning that appropriate treatment in newly diagnosed HF patients significantly ameliorated their inflammatory status after 12 months by reducing several pro-inflammatory mediators, which included TNF-a, IL-6, IL-1, C-reactive protein (CRP), and Vascular cell adhesion protein 1 (VCAM-1) [97].

### 4.2. Trimethylamine N-Oxide

Trimethylamine N-oxide (TMAO) is an active metabolite produced by the intestinal microbiota as the result of dietary choline, betaine, and L-Carnitin metabolism [111]. The bacterial populations of *Peptostreptococcaceae* and *Clostridiaceae* may raise the plasma levels of TMAO [112]. A growing number of studies have proven that increased levels of TMAO are strongly associated with the progression of HF stages and may affect the prognosis of cardiovascular disease. Abnormal concentrations of TMAO are correlated to advanced diastolic dysfunction of the left ventricle and may predict prolonged adverse outcomes in chronic HF [113]. TMAO may contribute to the deterioration of cardiac function through the following pathways: myocardial hypertrophy and fibrosis, endothelial and mitochondrial dysfunction, oxidative stress, and inflammation [114]. It should be noted that TMAO is responsible for the reduced expression of anti-inflammatory cytokines (e.g., interleukin 10 (IL-10)) and the overproduction of pro-inflammatory cytokines, such as TNF-α, interleukin 1β and 6 (IL-1b, IL-6), and transforming growth factor-β (TGF-β) [95,115]. A research by Seldin et al. revealed that TMAO may promote inflammation of the endothelium through the NF-κB pathway [94]. Furthermore, the activation of the NLRP3 inflammasome by TMAO enhances vascular and cellular inflammation, favoring pathologic cardiac remodeling and risk of ventricular arrhythmias [116,117]. Another interesting pathway proposed by Savi et al. suggests that mitochondrial impairment by TMAO is capable of altering normal myocardial function [118].

### 4.3. Short-Chain Fatty Acids

Short-chain fatty acids (SCFAs) are mostly produced by the gut bacteria and include acetate, propionate, and butyrate derived from the digestion of dietary fibers [119]. The *Bifidobacterium* species are mostly responsible for the production of acetate, which may affect the levels of butyrate and propionate secreted by the *Firmicutes* and *Bacterioidetes* populations [120]. Because they represent an energy source for the colonic mucosa, they may influence cell growth, gut motility, and proliferation in order to keep the intestinal barrier intact [96]. Apart from the absorption of sodium and water, short-chain fatty acids may alter the function of the immune and nervous systems, applying a potential anti-inflammatory effect [121].

Under normal circumstances, the myocardium covers its metabolic needs mostly through oxidation of long chain fatty acids (LCFAs) and, to a lesser degree, glucose, ketones, and lactate [122]. The failing heart muscle presents an impaired metabolic profile with reduced oxidation of LCFA and a decreased ability to produce SCFAs [123]. Patients with chronic HF have been found with decreased levels of SCFAs regardless of the LEVF [124]. The main reason for this metabolic rotation relies on the depletion of the gut bacteria producing SCFA, therefore resulting in deterioration of the SCFA’s cardio-protective anti-inflammatory potential [125].

Although the exact underlying mechanism is not yet elucidated, the role of SCFAs in cardiac function is pivotal. SCFAs may protect from HF through the following pathways: prevention of mitochondrial dysfunction, maintenance of normal blood pressure levels, modulation of the inflammatory environment through inhibition of histone deacetylases (HDACs), and moderation of inflammatory genes via G-Protein-Coupled Receptors (GPRs) [126]. The inhibitory effect of HDACs and NF-κB activity may restrain the rate of cardiac hypertrophy [127]. SCFAs exert an agonistic effect on GPRs by reducing the risk of increased left-ventricular end-diastolic pressures, perivascular fibrosis, arterial hypertension, and stiffness, as depicted in GPR41-, GPR43-, and GPR 109A-knockout mice fed normal chow [128,129].

Bartolomaeus et al. have revealed that the impact of the cardiac inflammatory response on hypertension levels may be mediated by regulatory T-cells (Treg) [130]. SCFAs may also lower the production of certain pro-inflammatory cytokines, such as IL-6 and IL-8, and inhibit the expression of intercellular adhesion molecule-1 (ICAM-1) and vascular cell adhesion molecule-1 (VCAM-1) [131].

It is remarkable that SCFAs, especially butyrate, can keep intact the intestinal barrier. Butyrate is capable of ameliorating the intestinal barrier by stimulating tight junction protein Claudin-1 transcription and the macrophage/WNT/ERK signaling pathway [132,133]. Consequently, SCFAs are beneficial for cardiovascular health, as they may stabilize myocardial metabolism, regulate inflammation, protect from left ventricular hypertrophy, and support the intestinal barrier.

### 4.4. Zonulin

Zonulin, also known as pre-haptoglobin 2, is a protein equivalent of the zonula ocludens toxin of *Vibrio cholera* [134]. It is responsible for the balance of the intestinal barrier, especially in the overweight population, and it is mostly produced by the liver and the intestine [135]. The continuous consumption of wheat favors zonulin secretion [136]. Auto-immune diseases, celiac disease, and diabetes mellitus are characterized by pathologic zonulin plasma levels [137]. It is regulated by the C-X-C chemokine receptor type 3 (CXCR3) and may control a protein complex referred to as tight junctions in order to reversibly moderate intestinal permeability [138]. As a result, translocation of bacteria and/or their products may follow in blood circulation. Endotoxemia is the consequence of LPS translocation from the intestine to systemic circulation, contributing to chronic inflammation through the activation of Toll-like receptors in HF patients [139,140].

Recent studies demonstrate that zonulin may be a novel mediator between the impaired intestinal mucosa and HF. According to Oliva et al., patients with COVID-19 had increased gut permeability markers of zonulin and serum LPS and were predisposed to thrombotic events [99]. Ahmad et al. revealed that patients with chronic HF of ischemic and non-ischemic etiology had a more profound inflammatory profile accompanied by increased levels of plasma C-reactive protein (CRP) and zonulin, which could affect the prognosis of cardiac systolic function and their functional capacity [98]. It is worth noting that statin treatment significantly induced the secretion of CRP and zonulin in patients with chronic HF and further reduced the physical performance of this population [141]. Perticone et al. suggest that although the HF population had elevated inflammatory biomarkers, the levels of zonulin were reduced and associated with the severity of HF [142]. Notably, chronic treatment with probiotics reduced zonulin concentrations, restricted sarcopenia, and improved the performance status of this population [143]. These findings imply the importance of zonulin as a contributor to the inflammatory process and a potential therapeutic target in the complex relationship between the gut and HF.

### 4.5. Amino Acids

Amino acids are another category of biomarkers mostly found in high-protein meals, and they retain a crucial role in the gut–heart axis. Bacterial enzymes mainly produced by *Bacteroides*, *Propionibacterium, Clostridium*, *Fusobacterium*, *Streptococcus*, and *Lactobacillus* are capable of dissolving protein substrates to amino acids [144]. Chronic HF patients often have reduced amounts of amino acids, which are associated with the severity of the clinical syndrome and left ventricular systolic function [145]. The heart’s dependence on amino acids is the consequence of an enhanced myocardial anabolic environment and the lack of cardiomyocyte energy [146].

The amino acid tryptophan, which is degraded to its major metabolites, kynurenine and indole-3-propionate, is a contributor to cardiovascular disease [147]. In particular, HF induces the secretion of elevated plasma levels of kynurenine independent from the left ventricular ejection fraction [148]. The kynurenine pathway is activated by inflammatory cytokines, such as interleukin-1β (IL-1β), tumor necrosis factor alpha (TNF-α), and Interferon gamma (IFN-γ) [100]. The overproduction of kynurenine pathway metabolites in patients with HF was associated with higher mortality and restricted functional capacity [149]. Similarly, increased levels of the ratio of the inflammatory markers kynurenine–tryptophan and kynurenic acid were strongly correlated with elevated risk of HF [150]. Recently, Wang et al. proposed that elevated serum concentrations of the kynurenine to tryptophan ratio (KTR), an inflammatory biomarker, was associated with poor prognosis in HF, especially with values of KTR more than 0.044 [101]. Interestingly, indoxyl sulfate (IS), another metabolite of tryptophane, moderated the expression of the NLRP3 inflammasome via the AHR/NF-κB pathway, raised the concentrations of numerous inflammatory cytokines, such as IL-1, IL-18, and TNF-a, and rendered the myocardium susceptible to fibrosis, hypertrophy, and left ventricular systolic dysfunction [151].

## 5. Possible Gut Microbiota Targeted Treatments

Because gut dysbiosis is capable of influencing the severity and progression of HF, the discovery and application of novel treatments could be effective against this pathology (Table 3). Intervention in the dysbiotic intestinal microbiome includes dietary modification, exercise training, as well as the use of antibiotics, pre/probiotics, and molecules binding various mediators (TMAO) and anti-toxins (anti-LPS).

### 5.1. Diet Modulation and Exercise Training

A Western diet rich in saturated fat but low in unsaturated fat is positively associated with anaerobic microorganisms, such as *Bacteroides* and *Bilophila*, highlighting the inflammatory potential of the intestinal microbiome in relation to cardiovascular risk and HF [157,158]. However, dietary modulation based on the Mediterranean regime remains the cornerstone of treatment of HF and amelioration of the gut’s microbial environment. The Mediterranean diet includes more whole grains, legumes, vegetables, fruits, and olive oil, accompanied by moderate proportions of fish, dairy products, and red wine and low consumption of red meat [159]. During a study period of 15 years, Strengers et al. have proven that compliance with the Mediterranean diet may lower the risk of HF, especially in the male population, measured using modified Mediterranean Diet Scores (mMDS) [160]. Accordingly, Kouvari et al. proposed that adherence to a Mediterranean diet had a beneficial effect on the prognosis of HF with preserved ejection fraction and C-reactive protein during a 10-year follow-up [152]. These findings imply that the Mediterranean diet is a safe and cardio-protective regime capable of ameliorating the inflammatory environment.

Exercise training is the second pillar of treatment of HF. According to the guidelines of the European Society of Cardiology (ESC), exercise is strongly advised, as it may ameliorate quality of life and functional status and reduce the risk of HF hospitalizations [1]. Engaging in sports activities, such as simple, daily, mild aerobic exercise, compared to a sedentary lifestyle, can have multiple benefits for intestinal health. A recent meta-analysis by Malandish et al. has proven the favorable influence of aerobic exercise on the overweight HF population by decreasing the inflammatory mediators TNF-α, IL-6, and high sensitivity CRP (hs-CRP) [161]. The study by Queipo-Ortuño et al. on animal models revealed that physical activity induced modification of the intestinal microflora through an increase in cardioprotective short-chain fatty acids and by affecting the hormones leptin and ghrelin [162]. Accordingly, Liu et al. discovered that exercise training not only had a protective effect on cardiac systolic function of post-myocardial infarction (MI) mice but also regulated certain bacterial populations, among which the most frequent were *Butyricimonas* and *Akkermansia* [163]. However, different forms of exercise and their multiple effects on the gut microbiome have not been adequately studied.

### 5.2. Probiotics and Prebiotics

Probiotics are defined as living microbes capable of modulating the gut microbiome and restoring intestinal balance. Clinical trials from the last decade have highlighted the protective effect of probiotics on HF. Treatment with probiotics containing *Lactobacillus rhamnosus GR-1* reduced the progress of post-infarction left ventricular remodeling and ameliorated cardiac function in terms of systolic and diastolic levels in mice [164]. Similarly, *Saccharomyces* improved systolic dysfunction in an HF population as well as renal function and inflammation in the short term [165]. According to Moludi et al., probiotic therapy after myocardial infarction attenuated cardiac remodeling by affecting certain molecular and echocardiographic parameters [153]. Karim et al. have proven the benefits of multi-strain probiotics on HF-induced sarcopenia by enhancing the physical performance of these patients and restricting oxidative stress and inflammatory process [143].

Prebiotics are mostly fibers acting as nutrients for bacteria, promoting their growth and metabolic activity in favor of the organism. The consumption of prebiotics, which mostly includes fruits, vegetables, and whole grains, remains controversial. Marquez et al. revealed that a high-fiber diet limited pathologic blood pressure indices, myocardial fibrosis, and left ventricular hypertrophy [166]. Although similar favorable effects were observed by Komatsu et al. concerning the aforementioned pathologies and inflammation, inulin induced elevations in plasma triglycerides in mice with metabolic syndrome [167]. However, the use of fiber or acetate was not capable of restoring cardiac function in mice with genetic predisposition to HF [168]. The limited evidence from clinical studies along with the variety and complexity of gut microbiota render the use of probiotics and/or prebiotics supplementary.

### 5.3. Antibiotics and Potential Targeted Inflammatory Treatment

Antibiotics may moderate the biodiversity of the intestinal microbiome and affect the inflammatory response. Recent studies have started to reveal a possible anti-inflammatory and cardio-protective effect of antibiotics in HF. In a retrospective case control study during a follow up of almost 20 years, it was found that certain antibiotic therapies, especially sulfonamide, trimethoprim, and macrolides, considerably reduced the risk of HF, whereas cephalosporins had the opposite outcome [154]. In parallel, administration of polymyxin-β and tobramycin not only decreased the overproduction of pro-inflammatory cytokines by monocytes in patients with HF but also improved endothelial function [169]. Likewise, minocycline decreased the levels of pro-inflammatory cytokines in rats with ischemic-induced HF, leading to amelioration of the echocardiographic parameters of systolic function and depression [170]. Although some antibiotics have shown a beneficial effect on the inflammatory process, their administration in the control of the gut microbiome is restricted by side effects resulting from their chronic use, the creation of resistant microbial colonies, as well as the risk of cardiovascular events, mostly among the elderly [171]. Therefore, rational and careful application of antibiotic regimens is recommended depending on the infectious disease.

Over the last years, novel experimental treatments, which target certain metabolites or inflammatory mediators, have been shown to potentially be promising. Wang et al. have proven that 3,3-Dimethyl-1-butanol (DMB), a choline analogue, inhibited the plasma concentration of TMAO and prevented the progress of left ventricular hypertrophy, fibrosis, and HF in animal models [156]. Ursodeoxycholic acid (UDCA), a secondary bile acid, has shown evidence of anti-inflammatory action and may provide support in the maintenance of the intestinal barrier [172,173]. It should also be noted that UDCA has decreased the arrhythmic burden and the atherosclerotic process in animal studies [174,175].

Short-chain fatty acids (SCFAs) derived from the consumption of a diet rich in fiber have a favorable impact on metabolic syndrome by regulating glucose and lipid homeostasis. Apart from their anti-inflammatory properties and inhibition of left ventricular hypertrophy, butyrate alleviated cardiac systolic dysfunction by enhancing mitochondrial activity in rat models [176]. Similarly, Jiang et al. suggest a potential protection from ventricular arrhythmias after myocardial infarction induced in rats [177].

Empagliflozin, an antidiabetic sodium–glucose cotransporter-2 (SGLT2) inhibitor approved for the treatment of HF, modulated the intestinal flora by increasing the cardio-protective short-chain fatty acid producing bacteria and reduced the toxic concentrations of glycochenodeoxycholate, cis-aconitate, and uric acid [178]. Additionally, it also has anti-inflammatory properties by decreasing the IL-6 levels in HF patients and enhancing myocardial function by restricting oxidative stress [179,180].

### 5.4. Fecal Microbiota Transplantation

Fecal microbiota transplantation (FMT) refers to a method of administering bacteria through stool transfer from a healthy organism to the intestinal tract of a patient; it is mostly used for the treatment of *Clostridium difficile* infection. One study analyzed the benefits of stool transfer to individuals with recurrent infections from *Cl. Difficile*, resulting in improvement of intestinal homeostasis [181]. Fecal microbiome transplantation to overweight pre-HF with preserved ejection fraction (HFpEF) animal models had a protective effect on cardiac systolic and diastolic function. As stated by Hatahet et al., the possible mechanism may be the induction of the short-chain fatty acid butyrate, which contributes to the degradation of toxic branched-chain amino acids [155]. However, evidence for this technique is insufficient in order to be established in daily clinical practice.

## 6. Prognostic Models for HF Based on the Gut Microbiome

The bidirectional relationship between HF and the intestinal microbiome emphasizes the need for algorithms diagnosing HF early, non-invasively, and accurately. Firstly, the group of Aryal et al. in 2020 demonstrated the dynamics of artificial intelligence based on a supervised machine learning model in distinguishing various intestinal microbial data from the American Gut Project and predicting the risk of cardiovascular disease (CVD), including HF, in a sample of 951 individuals [182]. Similarly, Hodzic et al. developed a predictive algorithm of atherosclerotic cardiovascular disease based on the distinction of certain gut microbial signatures from stool samples, attaining an area under the curve (AUC) score of 0.926 [183]. Furthermore, Erawijantari et al. from Finland present the FINRISK Microbiome DREAM challenge as a potential model for the prognostic risk of HF through localization of certain gut microbiomes, especially those associated with inflammation, during a 10-year period. This program included numerous forms of clinical evidence and microbiome data in a population cohort of over 7000 adults, thus facilitating the prediction of HF and the identification of high-risk patients [184]. Despite the small number of studies, the discovery of novel HF predictive models remains fundamental.

## 7. Conclusions

Gut dysbiosis and HF are two interrelated factors severely impaired by the presence of inflammation. Patients with HF have demonstrated significant alterations in the composition of the normal gut microbiome depending on the severity of the left ventricular ejection fraction. HF may disrupt the integrity of the intestinal barrier, resulting in the translocation of microbes and their metabolites, thus enhancing the inflammatory environment. In addition, the intestinal microbiome and inflammation are capable of inducing numerous pathophysiological pathways in which cytokines and various metabolites may have a serious impact not only on the prognosis but also on the progression of HF. Apart from diet modulation and exercise training, the rest of the therapeutic strategies rely on clinical studies in human and animal models. Consequently, the regulation of the intestinal microflora has become a promising field of research for the treatment of HF, as the discovery of new metabolic pathways may unfold more concepts for novel therapeutic interventions depending on the genomic profile of each individual.

## Figures and Tables

**Table 2:** Mediators contributing to the inflammatory process in the gut–heart axis.

Inflammatory Mediator	Source	Mechanism	Impact on Heart Failure	Key References
Lipopolysaccharide (LPS)	From Gram-negative bacteria	Activates NF-κB pathway, triggers innate immune response, promotes endotoxemia	Leads to myocardial apoptosis and fibrosis	Nguyen et al. (2023) [92], Asgharzadeh et al. (2018) [93]
Trimethylamine N-oxide (TMAO)	Produced by gut bacteria from choline, carnitine	Promotes inflammation via NF-κB pathway, induces mitochondrial dysfunction	Linked to myocardial hypertrophy, fibrosis, and poor prognosis	Seldin et al. (2016) [94], Tang and Hazen (2017) [95]
Short-chain fatty acids (SCFAs)	Produced by Firmicutes, Bifidobacteria	Maintain gut barrier, inhibit histone deacetylase (HDACs), regulate inflammation	Lower SCFA levels linked to impaired cardiac metabolism	Liu et al. (2021) [96], Modrego et al. (2023) [97]
Zonulin	Produced by gut epithelial cells	Regulates intestinal permeability, contributes to bacterial translocation	Increased levels linked to poor cardiac outcomes	Ahmad et al. (2022) [98], Oliva et al. (2021) [99]
Amino acids (tryptophan)	Bacterial breakdown of dietary proteins	Degraded into kynurenine; overproduction triggered by inflammatory cytokines	High kynurenine levels correlate with HF severity and mortality	Ala and Eftekhar (2022) [100], Wang et al. (2024) [101]

**Table 3:** Treatment strategies targeting gut microbiota.

Treatment Type	Mechanism	Benefits	Key References
Diet Modulation	Promotes beneficial bacteria (e.g., Mediterranean diet)	Reduces inflammation, improves gut barrier, lowers cardiovascular risk	Kouvari et al. (2023) [152]
Probiotics	Restores microbial balance; produces SCFAs	Reduces systemic inflammation, improves cardiac function	Moludi et al. (2021) [153]
Antibiotics	Modulates microbial diversity; suppresses harmful bacteria	May reduce inflammation, endothelial dysfunction	Loosen et al. (2023) [154]
FMT	Transfers healthy microbiota from donor to patient	Potential to restore gut balance; experimental	Hatahet et al. (2023) [155]
TMAO Inhibitors	Blocks production of inflammatory metabolite TMAO	Reduces cardiac fibrosis and inflammation	Wang et al. (2020) [156]
